# Editorial: Exosomes as Therapeutic Systems

**DOI:** 10.3389/fcell.2021.714743

**Published:** 2021-07-21

**Authors:** Bruna Corradetti, Deyarina Gonzalez, Inês Mendes Pinto, Robert Steven Conlan

**Affiliations:** ^1^Department of Nanomedicine, Houston Methodist Research Institute, Houston, TX, United States; ^2^Center of NanoHealth, Swansea University Medical School, Swansea, United Kingdom; ^3^Texas A&M Health Science Center, College of Medicine, Bryan, TX, United States; ^4^International Iberian Nanotechnology Laboratory, Braga, Portugal

**Keywords:** exosomes, extracellular vescicles, stem cells, exosome mimetic, nanomedicine, cell-free therapeutics, manufacturing, mimetics

For longer than 30 years, researchers from around the world have put a lot of effort in the application of nanotechnology to clinical therapy, in the attempt to maximize the effectiveness of any therapeutic agent while reducing its side effects. Through an inter- and multi-disciplinary approach, it has been possible to develop finely functionalized platforms able to specifically target cells of interest, temporally and spatially control the delivery of the agent while maintaining its bioactivity and immunotolerance. The goal of developing strategies with the potential to achieve all these functions at the same time, however, is still far from completion.

Advancements in the nanotechnology field have, on the other hand, allowed scientists to identify and characterize the natural delivery systems exploited by cells within our body to communicate, in physiological and pathological conditions. These nanoscopic particles, in the range of 50–150 nm, are called exosomes and provide a unique and multifaceted opportunity for the development of cell-free therapeutics. Exosomes are produced by all cell types and display similar properties as their parental cells, including their molecular composition and function. The use of exosomes offers several advantages over cellular-based therapeutics, as they easily penetrate across organs and tumor interstitium (*size advantage*), do not suffer from a by-stander effect thus maintaining their properties even in an immunosuppressive environment (*activation advantage*), and have a greater shelf-life, thus allowing for a long-term storage. Compared to synthetic formulations, exosomes display an inherent targeting specificity and a natural lipophilic core that compartmentalizes native materials and provides stability to the cargo. For these reasons, clinical trials testing the use of exosomes as therapeutic agents for the treatment of various diseases are currently ongoing. However, exosomal products have not yet been approved by the FDA, as scalability and standardization remain the major limitations for their broader use in clinical settings. In this special issue, we portrait the recent advances in the preparation, characterization and preclinical use of exosomes and exosomes mimetics for a better understanding of their therapeutic impact (Figure 1).

**Figure 1:**
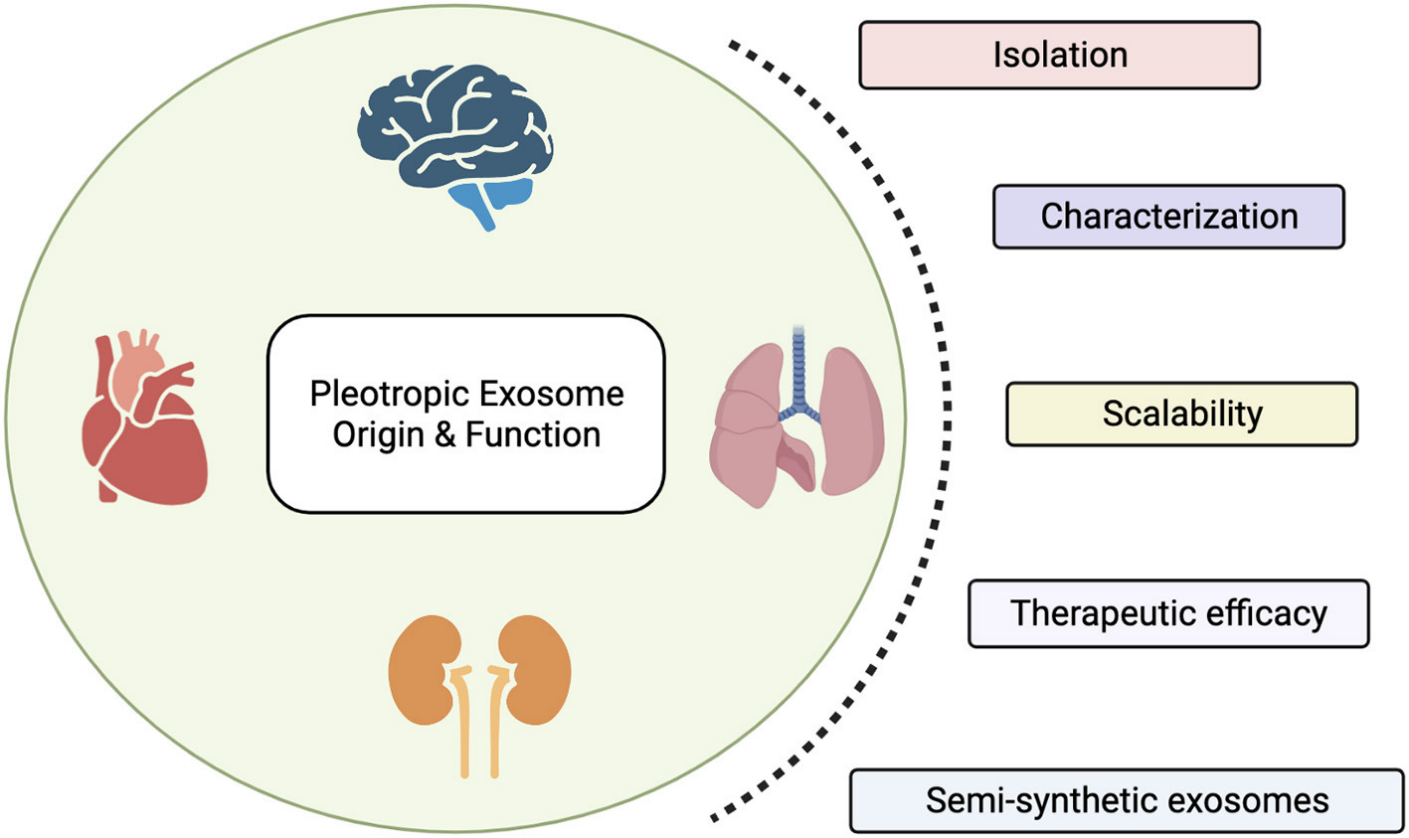
Exosomes are nanoscopic particles released by cells to communicate. Their molecular and biophysical properties change depending on their parental cell type, with biological implications in health and disease. In this special issue we highlight the therapeutic efficacy of exosomes and discuss the main limitations scientists working in the field encounter in their translation into clinic, including isolation and characterization procedures, and the need of a standardized approach to scale-up their production. Finally, we propose the production of semi-synthetic exosomes as novel approach to overcome those limitations and their opportunity to be conceived as reconfigurable cell-free.

Therapeutic intervention mediated by cells and their derivatives with immunomodulatory properties offers a broad range of applications spanning from regenerative medicine to oncology.

This collection includes preclinical studies testing the protective effect and therapeutic efficacy of exosomes from multiple cell sources for the treatment of chronic diseases.

Stem cell therapies are among the most promising types of treatment to combat the rising prevalence of chronic diseases. In fact, the number of clinical trials conducted with stem cells, particularly with mesenchymal stem cells (MSCs), has increased exponentially over the past few years. MSCs are adult stem cells that can be isolated from human and animal sources. They are self-renewable, multipotent, genetically stable, relatively ease to culture *in vitro*, and do not present ethical issues compared to embryonic stem cells. They exert a myriad of therapeutic roles in paracrine manner suggesting a key role for the extracellular vesicles (EV) they release in mediating their effects. Exosomes derived from stem cells are currently being explored as potential cell-free therapeutics with protective and immunomodulatory properties. In their review, Gowen et al., offer an overview of the current use of MSC-EV as an alternative to their cellular counterparts and discuss preclinical studies and current clinical trials for chronic diseases including diabetes, cardiovascular disease, and macular degeneration. The authors highlight current challenges around MSC-EV implementation such as large-scale manufacturing, characterization of EVs loading agents, pharmacokinetics, biodistribution, safety profiles, and the existing hurdles in translating the success obtained in preclinical studies to clinical settings. Nevertheless, the potential therapeutic benefit for patients merits the developing of MSC-EVs as therapeutic treatment options. Ramirez-Bajo et al. demonstrate that the effect of EVs derived from stem cells isolated from bone marrow and adipose tissue (BMSC and ATSC, respectively), display opposite effects, which also depend on the administration approach (Ramirez-Bajo et al.). Specifically, authors propose the autologous administration of exosomes isolated from BMSC as a potentially promising therapy to improve kidney graft outcomes in rats with chronic mixed rejection, while the allogeneic use of exosome from ATSC results in the exacerbation of the progression to end-stage kidney. In addition, when the role of BMSC derivatives (including conditioned medium and exosomes) in exerting a renoprotective effect is evaluated, only a partial recovery is found in mice with chronic cyclosporine nephrotoxicity following the treatment with exosomes, highlighting the requirement of an optimal regimen (i.e., dose and timing) to achieve a better long-term outcome (Ramirez-Bajo et al.). The broad-spectrum therapeutic capacity of MSC-EVs is further highlighted in the study by Kholia et al.. Here, the authors report the ability of MSC-EVs on halting kidney injury in a mouse model of toxin (aristolochic acid)-induced nephropathy, a disease featured by its strong fibrotic, inflammatory, and apoptotic components that ultimately evolve into carcinoma in 40% of the cases. In this context, intravenously administration of MSC-EVs were shown to significantly downregulate pro-fibrotic genes and improve kidney regeneration from toxin-induced injury. The ability of EVs to reduce fibrosis signaling at the genetic and protein levels has also been described in Liver regeneration. In a study from Li et al., EVs from multi-model normal hepatocytes were shown to promote anti-fibrotic actions while reducing the activation of major fibrosis-producing cells in the liver and their fibrogenic actions including inflammatory, immunological and proliferative molecular cascades. While these evidences reinforce the therapeutic potential of EVs in the regeneration of toxin-mediated fibrotic liver, it remains elusive our mechanistic understanding of how EVs can control fibrosis and potentially more severe forms of liver disease. Branscome et al. offer their perspective on the beneficial use of stem cell-derived exosomes in the treatment of an alternative approach to traditional cell-based therapies for the repair of cellular damage associated with pathologies associated to the central nervous system (CNS). EVs role in mediating physiological and pathological processes of the CNS have been widely explored given all cells of the CNS secrete exosomes. Interestingly EVs have been reported to exert bi-directional effects by either facilitating disease development or by providing protection against injury. Within the nervous system EVs have been found to support myelination, an important process that supports myelin formation and its function of increasing the speed of nerve impulses. Changes in myelination affect circuit function through life with demyelination associated with several neurodegenerative diseases, autoimmune diseases, and age-associated cognitive decline. In this special issue Domingues et al., provide a comprehensive review of the use of exosomes as diagnostic and prognostic biomarkers of demyelination associated conditions, with particular emphasis on exosomal biomarkers in multiple sclerosis (MS). Additionally, EVs-mediated mechanism of promoting *de novo* myelination including the therapeutic effect of EVs secreted from peripheral MSCs on myelination is elegantly discussed.

Boulestreau et al. offer their perspective on exosomes from stem cells as a suitable tool to obtain a comprehensive understanding of the effect of senescence in aging. In particular, this group suggests age-related alterations in the number and molecular/bio-chemical features of stem cells (inherited by the exosomes they produce) as the primarily cause of aging and propose their therapeutic potential to improve age-related diseases.

Exosomes isolated from immune cell types have been proposed as a suitable approach to not only to boost the anti-cancer immune response but also to create a pro-regenerative environment. In this context, Lai Tung et al. propose exosomes derived from regulatory T cells as a stand-alone personalized therapy to prevent transplant rejection, due to their inherent protective and anti-inflammatory potential. While the capability of exosomes to promote and support thrombin generation is widely recognized, underlining their relevance in hemostasis and thrombosis, the plasticity attributed to immune cells opens the field to the investigation on the effects of the microenvironment on the exosomes they release. Tripisciano et al. demonstrate that platelet-, red blood cell, and monocyte-derived exosomes exhibit an enhanced pro-coagulant potential which the authors found to be dependent on differences in their membrane composition, including the higher levels of phosphatidylserine.

In recent years, EVs have emerged as a ubiquitous and functionally diverse modality for transferring information between different cells but also between organisms of different species. In addition to their roles in normal physiology, EVs allow molecular shuttling from pathogens (e.g., Parasites) to hosts and serve as means for antigens and infection diseases spreading. In the elegant study by Zhou et al., exosomes isolated from Tick saliva and salivary glands are shown to inhibit wound healing as a potential mechanism to facilitate blood feeding and pathogen transmission while evading potential host immunological responses. At the molecular level this inhibitory effect underlies the downregulation of the host C-X-C motif chemokine ligand 12 and upregulation of interleukin-8, highlighting the importance of selective molecular targeting in the development of new therapeutic and vaccine strategies. The potential for extracellular vesicles derived from virus infected cells or extracellular vesicles carrying viral factors for therapeutic use was also explored by Dogrammatzis et al., who interestingly propose how extracellular vesicles may be hijacked and utilized by pathogens, and that this in turn could underpin the optimization of existing therapeutic tools and develop novel approaches.

In this special issue we also include manuscripts discussing the effect of the existing methods for exosome production, analysis and storage on their structure and function. With their work, El Baradie et al. address the challenge of storage and preservation that in most cases affect exosomes bioactivity and ultimately limit their clinical application. In particular, they compare the most used methods for exosome isolation (ultracentrifugation and ultrafiltration through tangential fluid filtration) and optimized the lyophilization method. The results obtained demonstrated that while exosome storage at low temperature determines a marked reduction in their integrity, by combining ultracentrifugation with a freeze/dried method in the presence of trehalose it is possible to retain size, number, and bioactivity of exosomes, facilitating their therapeutic applications. Recognizing the limitations of Nanoparticle tracking analysis (NTA) techniques commonly used to measure extracellular vesicles concentration in biofluids, Shearn et al. systematically investigated factors that could interfere NTA when looking at neat biofluids and proposed a robust methodological approach to address some of the limitations of this technique. Combined with other techniques such as Atomic Force Microscopy (https://doi.org/10.1039/D0NR09235E) the physical characteristics of extracellular vesicles can be carefully validated.

While significant progress has been made in the development of cell-free therapeutics, mimicking the composition and design of the natural tools our body uses in physiological and pathological conditions holds the promise to improve the outcome of biological devices and facilitate their translation into clinical settings. Exosomes offer a great opportunity for the improvement of current therapeutic systems in terms of payload tuning, targeting specificity and design, with a broad spectrum of applications, spanning from regenerative medicine to oncology, to infectious diseases. As such, exosomes are being conceived as reconfigurable therapeutic systems (including both, semisynthetic, and synthetic exosomes) with the potential to increase pharmaceutical acceptability through a controllable assembly process. In this special issue, the convergence that lies between natural and semi-synthetic exosomes is highlighted by the work proposed by Pisano et al.. To overcome challenging large-scale production of exosomes which is current limiting the translationability of naturally occurring exosomes in the clinic, the authors developed a reproducible and time-consuming approach to produce Immune (Cell) Derived Exosome Mimetics (IDEM). IDEM are formulated from monocytic cells by combining sequential filtration steps through filter membranes with different porosity and size exclusion chromatography columns. The approach utilized by this group allows for about 2.5-fold increase in the fabrication of semi-synthetic exosomes displaying similar physiochemical and molecular characteristics of their natural counterparts. Interestingly, IDEM show a higher encapsulation efficiency (over 30%) and drug release over time compared to natural exosomes, unraveling the advantage of IDEM in reducing side-effects while increasing cytotoxicity of targeted cells. In addition, the use of monocyte-derived exosome mimetics combines the advantages of a biomimetic drug-delivery system with reduced immunogenicity as they do not induce systemic inflammation following infusion nor face being rejected by the immune system. Along the same line, Chivero et al. show the manipulation of extracellular vesicles miRNAs as an efficient means for delivery of RNA-based therapeutics to target organs. In their study, the authors demonstrate that engineered extracellular vesicles loaded with miR-124 can effectively delivered this miRNA into the CNS, thereby alleviating cocaine-mediated microglial activation. Psychostimulants such as cocaine activate microglia by downregulating miR-124 resulting in neuroinflammation, and miR-124 restoration via extracellular vesicles delivery is effective at alleviating this cocaine-mediated neuroinflammatory effect in a murine model.

Finally, Killingsworth et al. minireview discusses the potential of liquid biopsies for translational opportunities reflecting on the different components of liquid biopsies including exosomes. The authors briefly explore the relevant role of EVs as biomarkers of disease, within this platform, while discussing EVs therapeutic potential with challenges around EVs heterogeneity and dosage marking the future directions for EV therapeutics.

By combining key properties of natural exosomes with those associated to synthetic nanoparticles it is possible to improve manufacturing scalability and cost-effectiveness, enhance drug loading efficiency and targeting, and increase bioavailability while preserving organotropism. The possibility to develop novel reconfigurable cell-free therapeutics for the treatment of a broad spectrum of degenerative diseases through a personalized approach is currently attracting the interest of national and international Biotech companies. The global market for exosome therapeutic is expected to reap the revenue of $582.45 million by 2027.

## Author Contributions

BC and RC wrote the article. DG and IM have made a direct contribution to the work. All authors have approved the article for publication.

## Conflict of Interest

The authors declare that the research was conducted in the absence of any commercial or financial relationships that could be construed as a potential conflict of interest.

